# Analysis of Grain Size and Distribution in Fe-B-C Alloy Using Optical Microscopy and Image Analysis

**DOI:** 10.3390/ma18030596

**Published:** 2025-01-28

**Authors:** Lenka Křivánková, Rostislav Chotěborský, Barbora Černilová, Miloslav Linda

**Affiliations:** 1 Department of Material Science and Manufacturing Technology, Faculty of Engineering, Czech University of Life Science Prague, Kamýcká 129, 165 00 Prague 6-Suchdol, Czech Republic; 2 Department of Electrical Engineering and Automation, Faculty of Engineering, Czech University of Life Sciences Prague, Kamýcká 129, 165 00 Prague 6-Suchdol, Czech Republic; cernilova@tf.czu.cz (B.Č.); linda@tf.czu.cz (M.L.)

**Keywords:** Fe-B alloys, cell size, microstructure measurement, image analysis

## Abstract

The size and morphology of the grains of a material and their distribution have a significant impact on the mechanical properties of the material (and their further application). Based on the data obtained from image analysis, it is possible to modify the microstructure of materials. Within the formation of a eutectic, borides occur along the austenite grain boundary. The cell size can be managed by technological process (forming) or by adding chemical elements. In this paper, a method of measuring the cell size of a hypoeutectic Fe-B-C alloy across the entire examined cross-section of the sample was researched by creating a mosaic from individual frames. Sample preparation allowing clear grain boundary visibility was essential. It was observed that the most effective results were achieved with quenched microstructures etched using Klemm I color etchant. A Zeiss optic microscope with AxioVision software (AxioVision SE64 Rel. 4.9.1.) was used for image acquisition, and mosaics were created using MosaiX software. This study revealed that, before further processing, images must be segmented to address color inconsistencies using average grayscale values. This preprocessing step enabled precise cell size analysis through an algorithm implemented in Scilab. The developed methodology was used to create sample maps for determining the grain size and its distribution in the Fe-B alloy. This automated approach provides a comprehensive dataset, enabling detailed analysis of both individual images and the entire sample. Manual grain size measurements were performed for verification, and statistical analysis demonstrated a close correspondence between the results. The results confirmed a significant impact of the added alloying elements on microstructural homogeneity in hypoeutectic Fe-B-C alloys. Homogeneity decreases with the addition of alloying elements such as chromium and vanadium, while tungsten contributes to a more stable grain size. A low gradient value shows small grain size changes from the core to the edge in the cross-section. Furthermore, the results show that higher amounts of chromium increase the average grain size values. The results demonstrate that automated methods allow for comprehensive analysis of the entire sample, enabling precise determination of grain size and other properties across the entire object rather than only on subjectively selected areas. This approach effectively eliminates the influence of human error, ensuring more reliable and consistent data.

## 1. Introduction

Metals and their alloys are widely used materials in various industries. The growing demands to reduce manufacturing and operating costs along with improving the properties of materials are increasingly challenging for materials engineers. One of the most problematic aspects of this industry is the issue of tool wear. Wear, especially abrasive wear, is particularly evident in industries such as agriculture, earthworks, or mining, where the problem arises when soil particles interact with tools such as shares, chisels, or crushers [1]. More and more attention is paid to the production of alloys containing boron for their high resistance to abrasive wear accompanied by a low price [2,3,4,5,6]. Boron forms borides in the microstructure, which are characterized by high hardness, wear resistance, and toughness. Iron alloys with a boron content (0.5 to 4.0 wt. % B) reduce the economic demands and consumption of traditional alloying elements (Cr, Ni, V, Ti, etc.) [7]. As little as 0.001 wt. % B leads to an increase in the hardenability of steel. Borides are used as a hard phase for iron alloys, where the borides form a support structure that is resistant to wear, especially in Fe–Cr–B and Fe–B–C alloys [8,9]. There are new possibilities and the assumption that borides could replace carbides in the future, as compared to carbides, borides offer several advantages, including greater hardness, thermal stability, and Young modulus. Recent studies have identified metal borides, particularly Fe_2_B, as potential strengthening phases [10,11]. However, there is only limited experimental work available on the nanomechanical properties of Fe_2_B and even less on the FeB phase as precipitating phases in boride-alloyed materials. In addition, various scientific groups have recently shown interest in studying the effect of ligation on the mechanical properties of hard boride phases. Research has shown that properties can be significantly influenced by alloying elements, but their individual influence has not yet been well defined and fully understood [10,12,13].

Current research shows that the most important factor influencing wear resistance is not hardness, but chemical composition, microstructure type and morphology, and the ability to form secondary protective phases. The grain structure is therefore a key investigated property for evaluation by metallographic techniques and the basis for the creation of new materials with special required properties. The detection of microstructure is typically performed using X-ray analysis, optical microscopy, or scanning electron microscopy [14,15,16,17]. Two-dimensional and three-dimensional object detection and their subsequent classification are among the processes in which various properties such as object edges, primitive shapes, and color scale are distinguished using image and video recordings [18]. Due to the difficulty of detecting microstructural features in their volume, those parameters are usually determined using a two-dimensional cross-sectional image of the observed sample [19]. Average values of grain size can be determined in several ways, both manually and automatically [19]. One of the basic manual options is drawing a straight line and then dividing the length of the line by the number of grains that are within it [20]. Manual methods are time-consuming and subject to random errors and suffer from poor repeatability in estimating microstructural features such as grain boundary length, grain size, and grain number [21,22]. Nowadays, automatic techniques are commonly used, which are defined by ASTM standards, and various software can help to evaluate them [23]. Attention is currently being paid to the evaluation of microstructural parameters using machine learning [19]. Jung et al. (2022) [19] evaluated the average grain size from images using machine learning by applying a convolutional neural network, which made it possible to determine the boundaries of individual grains and subsequently determine the grain size with high accuracy. Within the microstructure analysis, the threshold is the essential parameter for determining grain size, which allows separating individual cells from boundaries [23]. A threshold can be applied automatically. However, this requires a prior analysis of the grayscale distribution of each frame. This can be done using a gray level histogram [24]. As part of this study, automatic methods will be used to determine the size of cells from the microstructure of the samples. A lot of attention has already been given to these methods; however, that attention has been given to the analysis of individual images with unchanging chemical composition [23].

The aim of this study is to develop and validate a reliable method for grain size measurement (determined as grain surface area in μm^2^) in hypoeutectic Fe-B-C alloys, with a particular emphasis on efficient processing of large datasets from image analysis. This involved the development of a novel algorithm. Unlike traditional methods that analyze individual images, this study innovatively utilizes a large dataset (eight samples, up to 750 images per sample) for comprehensive microstructure analysis and quantification of its variability. The results, validated by correlating automated and manual measurements, provide a deeper understanding of the relationship between material composition and properties. Another objective is to determine the grain size and its distribution in eight samples of hypoeutectic Fe-B-C alloys with varying chemical compositions.

## 2. Materials and Methods

### 2.1. Sample Preparation

Ferroalloys and steel bars were used to produce steel batches for casting in this study. EDS analysis on a (Tescan MIRA3 GMU, Brno, Czech Republic) scanning electron microscope was used to determine the content of elements in ferroalloys shown in Table 1. From Table 1, it can be seen that four types of ferroalloys were used to create the samples. Specifically, these were FeB (ferroboron), FeCr LC (low-carbon ferrochrome), FeW (ferrotungsten), and FeV (ferrovanadium). These alloys were used because of their specific elements (B, Cr, W, V) that can affect the properties of the final steel alloys. There was a balanced amount of iron within the chemical composition.

Steel bars were used as the base material, which was further combined with ferroalloys to produce alloys. The chemical composition of steel bars was analyzed by a Bruker Q4 TASMAN optical emission spectrometer (Bruker AXS SE, Karlsruhe, Germany); the results are listed in Table 2. The table shows that the steel used was C35 steel.

C35 rolled steel was used as the base material. The steel bars were machined on a lathe to remove the scale. A total of eight samples of different chemical compositions were prepared and named K1 to K8. These samples were produced as a combination of the respective ferroalloys and C35 steel. The chemical composition (obtained by OES analysis) of each sample is given in Table 3.

**Figure 1:**
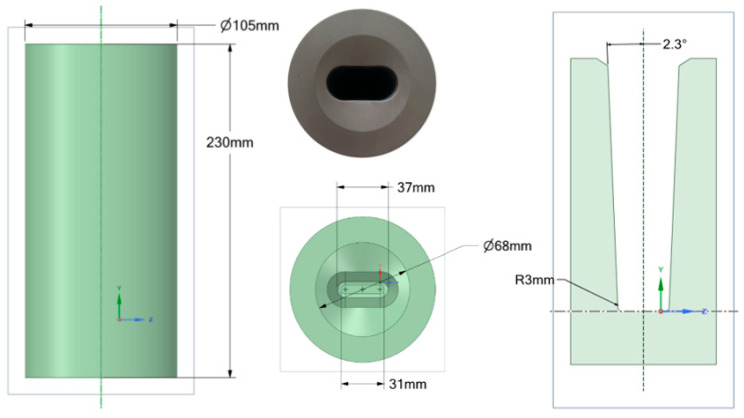
The graphite mold used for casting samples with different chemical compositions.

A vacuum medium-frequency induction furnace Indutherm VTC (Indutherm Erwärmungsanlagen GmbH, Walzbachtal, Germany) 200 V with a 1000 g effective ceramic crucible volume and power rating of 15 kW was used to cast the samples. The samples in this study were cast into a graphite mold with a modified shape and dimensions, as shown in Figure 1, considering the ease of creating samples for abrasion resistance. The graphite mold was preheated to 160 °C to absorb surface moisture and prevent damage to the furnace. After the batch was melted and mixed thoroughly, the smelted solution was poured into the graphite mold at 1650 °C. Elliptic cylindrical ingots with dimensions of 150 mm × 37 mm were obtained, followed by air cooling to room temperature.

All test samples were machined from the lower parts of the cast ingots using a Struers Discotom-10 cut-off machine. DuroCit Powder casting resin mixed with Durocit Liquid I and II was used to create the metallographic samples. This was followed by the preparation of the metallographic sample in sub-steps according to ASTM E3 [24]. After the resin cured, the sample was ground with a diamond wheel (Struers—MD Piano 220) with water for 5 min. This was followed by grinding with a diamond wheel (Struers—MD Alegro) with a 9 μm water-based diamond suspension for 4 min. The next step was to polish the sample with a wheel (Struers—MD Dac) with a 3 μm water-based DP-Dac diamond suspension for 4 min. The last step involved polishing the sample with a wheel (Struers—MD Chem) with a colloidal OP-S Al_2_O_3_ 0.05 μm suspension for 20 s. The clamping force was set to 20 N.

Following this step, every sample was thoroughly cleaned with water and dried properly to remove all impurities, namely polishing paste or suspension (particles of aluminum oxide, silicon dioxide, or diamond suspension), microscopic metal particles (small loose metal particles that have separated from the surface of the sample during polishing), lubricant (residue from the abrasive media used), dust, and solid particles (particles from the surrounding environment that have settled on the surface during handling), and make the sample perfectly clean. Prior to microscopic analysis, the sample was thoroughly cleaned and dried to ensure that the surface was clean so as not to distort the observed structure. The procedure involved rinsing the sample with demineralized water using an ultrasonic bath, rinsing the sample with isopropyl alcohol to remove grease, and drying the sample with a stream of dry air.

**Figure 2:**
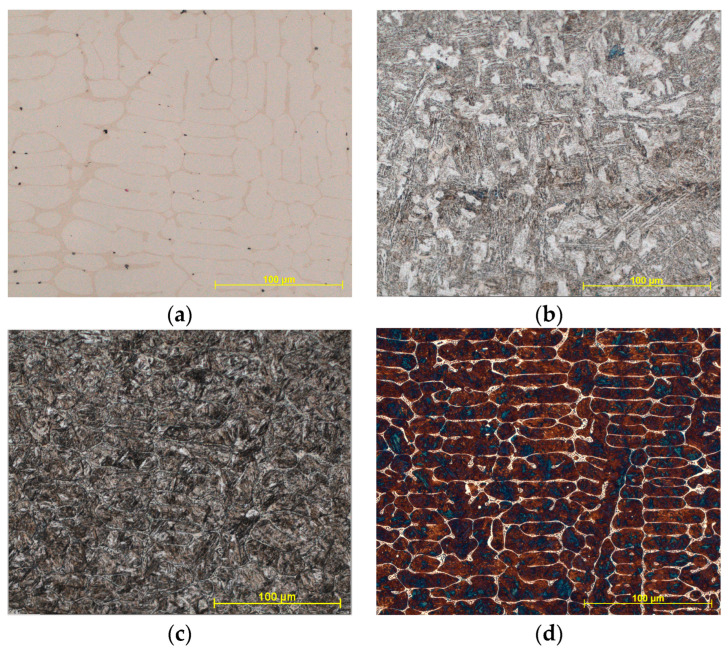
The samples were treated with different etching methods: (**a**) unpolished structure; (**b**) etched microstructure with Nital 2% etchant; (**c**) etched quenched microstructure with Nital; (**d**) etched quenched microstructure with Klemm I color etchant.

Inspection of the prepared samples under an optical microscope revealed the presence of borides, appearing as darker areas at the grain boundaries. However, the contrast was not sufficient, as evident in Figure 2a. To show the microstructure of the metals, it was necessary to etch the sample, so the common etchant Nital 2% (solution of nitric acid in ethanol) was used. The use of this etchant caused insufficient contrast for phase separation of borides, ferrite, pearlite, or other microstructure of the matrix after casting, as evident in Figure 2b. For this reason, the samples were quenched to form only a two-phase structure (martensitic with visible borides at the boundaries). The samples were placed in an oven set to a quenching temperature of 880 °C and quenched for 20 min. Then, the samples were cooled in a water bath. However, the etching of quenched samples with Nital 2% showed a weak RGB interface between the borides (whitish-yellow color) and martensite (ochre to brown color), as can be seen in Figure 2c. Therefore, Klemm I, coloring etchant (K_2_S_2_O_5_ + supersaturated liquid solution of Na_2_S_3_O_3_ in water) was used, which allowed the formation of sharp boundaries between borides and martensite. The borides were represented by white, and the martensite by brown to blue color. The microstructure of the Klemm I-etched sample is shown in Figure 2d. Etching with Klemm I etchant was then performed on all eight samples.

### 2.2. The Visualization of the Microstructure and the Matrix Creation

**Figure 3:**
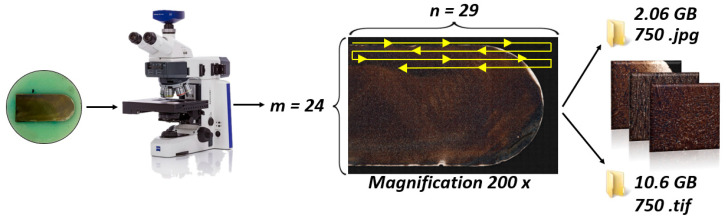
Process of creating a MosaiX image for the K6 sample. The yellow arrow indicates the direction of image acquisition (movement of the microscope camera while scanning the sample).

An optical microscope (OM, Zeiss Axiocam 305 color, Carl Zeiss Microscopy Deutschland GmbH, Oberkochen, Germany) with AxioVision software was used to visualize the microstructure. The microstructure was evaluated on one-half of the metallographic specimen to maximize the possible range of the microscope stage. The samples were imaged at 200× magnification, which was sufficient for the purposes of analysis. First, the large-area-image acquisition module, MosaiX, was used. Using the MosaiX module, it is possible to scan the sample one frame at a time and then combine the individual frames into a single “mosaics image”. A grid of array size (*m* × *n*) was created. Figure 3 shows the whole process of creating a mosaic for the K6 sample. From the figure, the direction in which the individual frames were taken is clear. The samples were also numbered according to the direction in which the frames were taken. The acquired frames were saved in *.zvi, *.jpg, and *.tif format. The *.zvi format is a proprietary file format created by Zeiss for storing image data from their microscopes. This format allows for the storage of not only images but also metadata. For example, a mosaic containing 29 columns and 24 rows was created for the K6 sample. This mosaic contained a total of 696 frames. The dimensions of one frame were 684.4 × 571.1 μm. At a resolution of 2464 × 2056 px, 1 μm corresponded to 3.6 px. The unevenness of the sample surface was reduced using the Auto Focus Correction option. Correct alignment of the tiles to each other was performed using the Stitching setting with a 10% overlap. The search depth was 15.

Since the etched surface area of each sample is on the order of cm^2^, uneven etching of the surface can be expected over such a large area. Due to the impossibility of uniform manual etching, some parts of the sample appear noticeably darker, while others are lighter. This is caused by the deposition of a sulfide film (40–500 nm) on the sample’s surface. The colors produced by the etchant are caused by interference and are visible under bright-field illumination. It is also important to note that each phase colors at a different rate. Generally, softer materials or materials less resistant to etching (ferrite) are etched more quickly than harder or more resistant materials (martensite). It is important to acknowledge that the manual etching process inherently introduces certain variations, which, in this instance, are deemed tolerable for subsequent experiments and analyses. It should also be pointed out that such a large photographed area may cause blurring, especially at the resin/alloy interface. Here, optical blurring occurs when capturing details at the boundary of these materials. This effect may affect the sharpness of the images in these areas but is still considered an acceptable phenomenon for analysis purposes.

### 2.3. Gray Scaling

**Figure 4:**
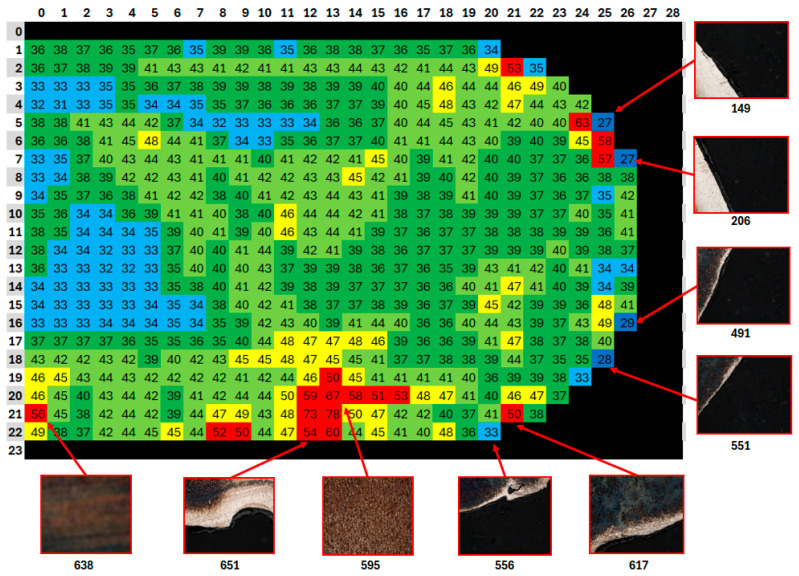
Detection of defect locations in the sample caused by flaws in the preparation of the etched surface and technological defects of the casting. The color assignment of individual frames is specified in Table 4.

For each mosaic image created, files from AxioVision were exported to determine the densitometric minimum values, densitometric mean values, and densitometric maximum values. These values corresponded to the minimum, mean, and maximum grayscale values represented in each frame. The grayscale value is the level of brightness represented by a given pixel. Due to the uneven etching and surface color changes caused by the etchant, the mosaic images were grouped based on the color spectrum and corresponding grayscale values. The grayscale values are shown in Figure 4, where they are specified by a numerical value. For example, the values for sample K6 are shown. The lowest average grayscale value for sample K6 was 21.65, and the highest was 78.13. The values in this interval were divided into seven groups, which were color-coded. The color difference is shown in Table 4 and was individual for each sample. This approach reduced the distortion during thresholding at the boride–matrix interface, thus minimizing the risk of misidentifying boundaries. It also enabled the detection of defects such as cracks or areas that were under- or over-etched, which were excluded from the analysis. Frames assigned the color black were excluded from the assessment, as they were frames of the resin or boundary frames between the resin and the sample, where the resin predominated in area. For sample K6, this included frames with an average grayscale value of up to 25. Additionally, frames assigned the dark blue color, corresponding to an average grayscale value greater than 30 and less than or equal to 35, were also excluded. In this case, these were boundary frames between the resin and the sample. These frames are indicated in Figure 4 with labels 149, 206, 491, and 551. Frames 149 and 206 clearly show a strong light boundary between the resin and the sample. The light color in the frame corresponded to the unetched part of the sample. Unetched areas of the sample were also noticeable in other parts and were excluded from the evaluation as well. These frames were assigned the color red for the final assessment. For sample 556, with an average grayscale value of 33.06, which corresponded to light blue, it was also excluded from the evaluation. From the figure, it is clear that a large part of the image consists of resin, and the results would therefore not be relevant. Furthermore, frames that were blurred due to microscope shifts were also discarded (638, 595).

A *.csv file was created to define the criteria, listing threshold grayscale values obtained during the configuration of each procedure. For example, for sample K6, the grayscale values were set to range from 30<procedure 1 ≤35, 35<procedure 2 ≤40, 40<procedure 2 ≤45, and 45 <procedure 4 ≤50.

### 2.4. Setup Procedures Within the AxioVision Program

**Figure 5:**
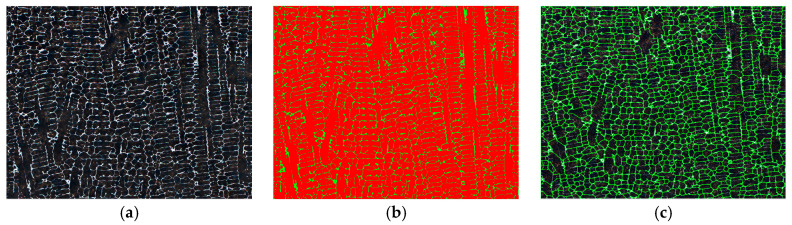
Measured image (**a**); Segmentation (**b**); Automatic Object Separation (**c**).

For each group from the color distribution according to the corresponding grayscale value presented in Table 4, a separate “procedure” was established. In this context, “procedure” means the sequence of steps set up in AxioVision. Within each color group, one frame was selected that roughly corresponded to the average grayscale value of the respective color. For sample K6, this selection was made for all groups except for black, dark blue, and red, as evident from Table 4. In total, there were 4 procedures. The procedures were set within the tree structure of AxioVision in the Program Wizard of the Automatic Measurement Program. This program can be used to measure any number of images using the Automatic Measurement Programs (Run function). The Program Wizard includes Measurement Program Management, where it is possible to set a new procedure or duplicate or delete an existing one. In setting up these procedures, it was first possible to adjust brightness, contrast, and gamma value; sigma filter; shading correction; and edge enhancement—these elements were left at the default settings of AxioVision and were not changed for any procedure. The adjustment was made during the Segmentation step, where the phases were divided as thoroughly as possible (see Figure 5). During this step, RGB depth settings are established to achieve the greatest contrast between the dark (matrix—Phase 1) and light (borides—Phase 2) areas of the individual frames. If only one RGB depth setting was used for all frames, not all cell boundaries would be rendered, and the resulting simplified images would not have the correct values indicating the cell size.

The next step after Segmentation is Deletion of Artifacts, Filling of Holes [25,26,27]. Here, the default settings of AxioVision were again maintained. This was followed by Automatic Object Separation. In this step, it was possible to choose either the Erosion/Dilation mode or Watersheds. After reviewing the more suitable mode, Watersheds, with a tolerance of 12 was selected. No objects were removed or added. In the Set Measurement Properties, the items we wanted to measure were Image Name, Image Phase Name, Densitometric Mean Value, Densitometric Minimum Value, Densitometric Maximum Value, and Area Frame. After this step, the data files were created. The data for each procedure were saved in the Flds and Regs files separately. Furthermore, criteria *.cvs files that contain grayscale thresholds were created. The Regs file contained specific information for each individual frame of a given sample; i.e., it contained how many cells and borides were on each frame, as well as information about their size. The Flds file contained overall information for each frame, one by one; i.e., the number of rows of this file corresponded to the number of frames of the respective mosaic.

### 2.5. Verification of Results by Manual Measurement

**Figure 6:**
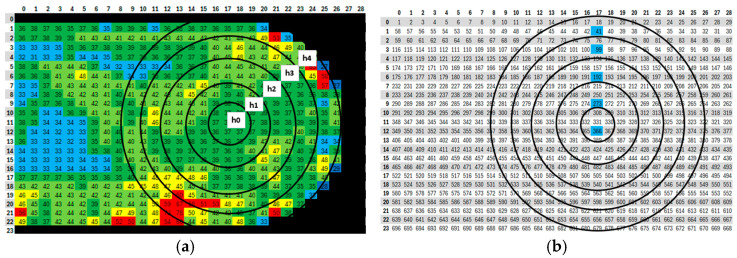
Sections of sample on which manual cell measurements were performed; (**a**) indicated depths h_0_ to h_4_. The color assignment of individual frames is specified in Table 4; (**b**) locations of the analyzed frames.

In order to ensure the accuracy of the cell size data in the alloy, manual measurements were performed, which allowed the establishment of upper and lower limits on the cell sizes. These limits were then used to remove erroneously detected cells from the automated measurements. The quality and reliability of the automated process results were improved by this procedure. These upper and lower limits were denoted by A min and A max. Manual measurement was conducted using AxioVision software, specifically the Outline spline function, which allows for accurate cell bounding. The area of each cell was expressed in mm^2^, with 1 mm^2^ corresponding to 13.15 px^2^. Verification was performed on five frames for each sample, with frames selected sequentially from the center of the sample towards the edges (depths denoted h_0_ to h_4_). The locations of the individual depths (h_0_–h_4_) within the mosaic are shown in Figure 6a. The locations of the individual analyzed frames are shown in Figure 6b. A minimum of 100 measurements were taken on each of these frames. This meant for each sample, a minimum of 500 cells were manually measured from the center to the edge of the sample. And this was done for all eight samples. The sample was divided into five band sections, with the depth marked as h_0_ corresponding to the center of the sample, and then frames were selected for the sections towards the edges of the sample. The specific frames from each area of the sample were as follows: Section h_0_: frame 366 with an average grayscale value of 37.36. Section h_1_: frame 273 with an average grayscale value of 38.07. Section h_2_: frame 192 with an average gray value of 41.13. Section h_3_: frame 99 with an average grayscale value of 44.02. Section h_4_: frame 41 with an average grayscale value of 35.11.

### 2.6. The Measurement Approaches

Three different measurement approaches were used to assess cell size. Measurements were conducted separately for each sample, selecting frames considered optimal for grain size evaluation. A total of eight material samples were used in this study. For each sample, five frames were manually measured at different depths (h_0_ to h_4_), and the pitch between frames was twice the frame height at a given magnification. Thus, a total of 40 frames were measured manually. Cell sizes in frames at depths h0 to h4 were obtained using “Manual Measurement”, with at least 100 cells measured in each frame. The Automatic Measurement 1 was performed with AxioVision software on the same frames on which the automatic measurements were performed. This automatic process generated a larger amount of data than the manual measurements because the software measured all cells in a given frame. To minimize errors in the automatic measurement, the values from “Auto 1” were bounded based on the values obtained manually. This means that the minimum and maximum values of the manually measured cells were determined. Values of the automatic measurement that exceeded this limit were excluded. This procedure ensured that the automatic measurement results were consistent with the manual measurements, thus eliminating potential errors caused by incorrect detection by the software. Using Automatic Measurement 2 (Auto 2), values were also obtained using AxioVision software, but this procedure included values bounded by maximum and minimum cell size values obtained manually from all 5 frames at all depths. Values from the automated measurements that exceeded this limit were again excluded.

### 2.7. Algorithm for Evaluating the Size of Cells on Individual Frames

**Figure 7:**
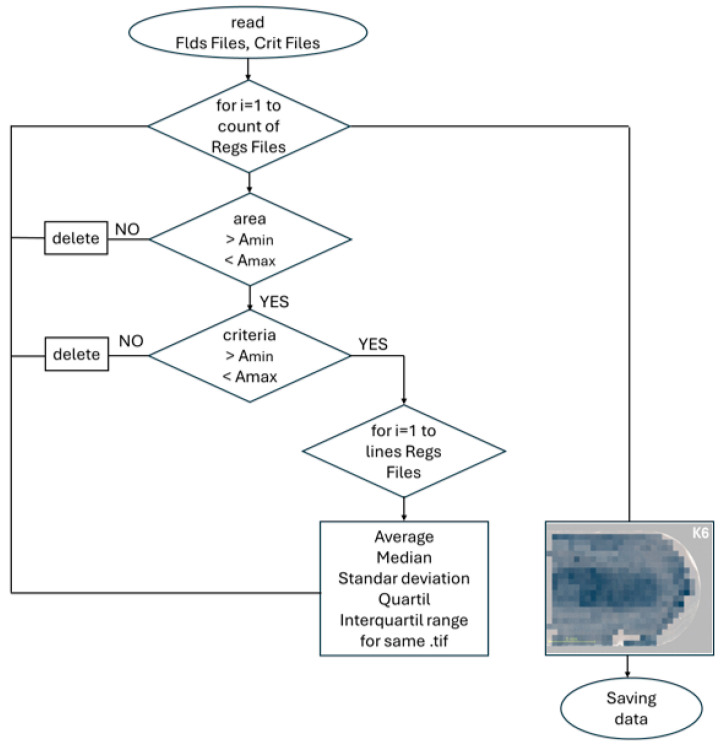
The flowchart of the data processing script for the analysis of cell size and distribution.

To create the matrix, an algorithm was created in SciLab in the SciNotes editor. The goal of the algorithm was to substitute individual microscope frames with a simplified image for further data analysis. The flowchart of the data processing script for the analysis of cell size and distribution is shown in Figure 7. Before running the algorithm, it was essential to define the number of rows *m* and number of columns *n* in the mosaic. Consequently, the lower and upper size limits for pixel clusters were defined. Only pixel clusters with areas between A_min_ and A_max_ were considered. An upper grayscale limit was set to a value of 225, corresponding to the maximum possible result value. The first step of the algorithm involved loading *.csv files. The input files included Regs, Flds, and a criteria file. The algorithm first identified rows in the Flds file with average grayscale values that matched the selected criterion from the criteria file. Grayscale values in the Flds file were compared to those in the criteria file, where the algorithm sought mean grayscale values greater than the lower threshold for the procedure in the criteria file. Filtering was implemented using a For loop, and rows with grayscale values matching the criterion were stored in a variable (thus, in the new file with filtered data). Subsequently, rows within this variable (created file) were filtered to find average grayscale values lower than the upper limit of the criterion for the procedure. After filtering, the reduced data were again stored in a new variable with newly filtered data. Data in this variable were further reduced by excluding rows that corresponded to Phase 2, which removed pixel clusters corresponding to borides and left only the matrix (Phase 1). Consequently, the Regs file corresponding to the criterion was filtered, beginning with the Regs file exported during the first procedure. Noise filtering was performed within the Regs file, removing pixel clusters outside the defined limits A_min_ and A_max_. Rows that met the conditions were stored in a new variable (created file), which was further filtered to exclude rows corresponding to the second phase. The algorithm then scanned through the newly created variable, searching for rows (clusters of cells) whose names corresponded to *.tif files with an average value matching the specified criterion. The *.tif file names were obtained from the reduced Flds file data. The data were then sequentially matched with individual frames, from the lowest to the highest in order.

## 3. Results

The mosaics were created for each of the eight samples with varying compositions. In Table 5, information about the resulting mosaics of individual samples is provided. The table includes the number of rows (m) and columns (n) in the mosaics, the total number of frames in the mosaics, the size of the resulting *.zvi file in kB, and the image width and height of the mosaics in pixels [px]. It is obvious that the file size increases with higher magnification; for sample K3, whose mosaic contains 638 frames, the total size of the *.zvi file is 18,957,788 kB at a magnification of 200×. The same sample scanned at 500× magnification had a file size of 117,133,848 KB. Therefore, higher magnification places more demands not only on the storage system capacity, but also on the computing power and time consumption of the whole capture process.

**Figure 8:**
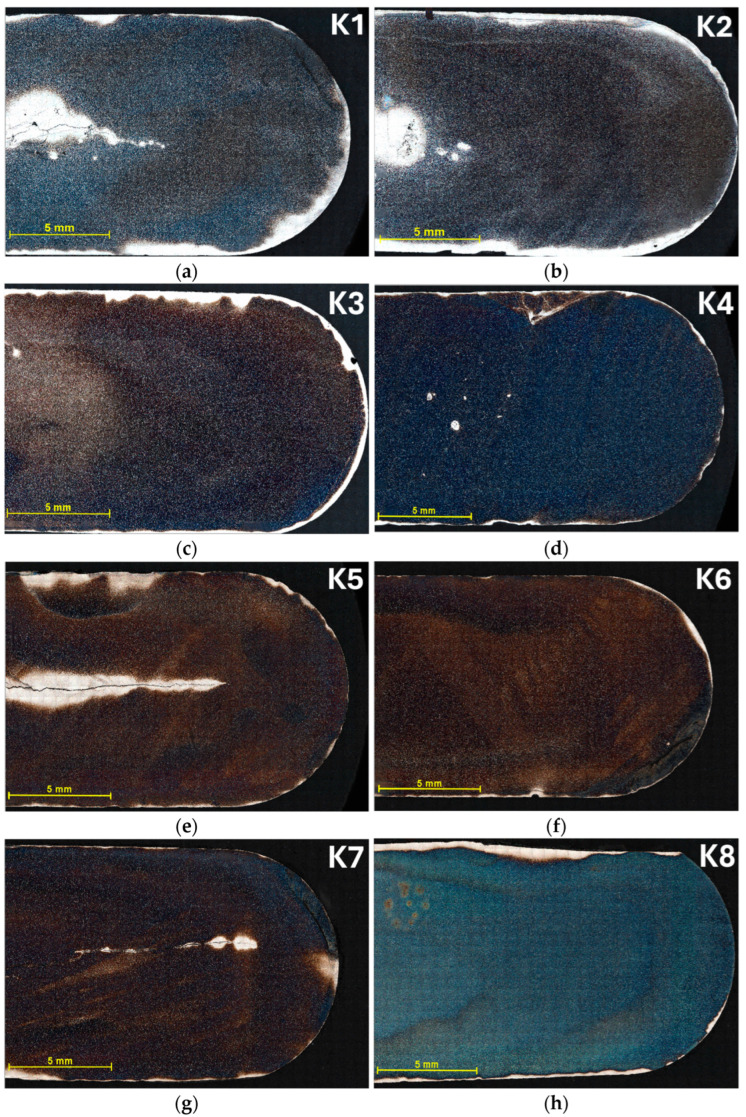
Mosaics (assembled images) of the tested samples from K1 to K8 obtained by MosaiX. The images are labeled (**a**–**h**), corresponding to the sequential numbering of the samples from K1 to K8, with K1 represented by (**a**) and K8 by (**h**).

In Figure 8, mosaic images are shown for all samples K1 to K8, stored in *.zvi format. Some of the samples display defects; for instance, cracks are visible in samples K1, K2, K5, and K7. These cracks occurred during the casting of the samples and their subsequent rapid cooling. In addition, bright areas are visible at the interface between the sample and the resin. These may be due to oxides forming in this area. Although the cracks and bright spots do not affect the overall results of the cell size analysis, they lead to data loss. This occurred because the structure near these cracks was poorly etched by the Klemm I etchant. Given the adequate sample size and the availability of remaining data, this particular loss does not significantly impact the overall evaluation.

**Figure 9:**
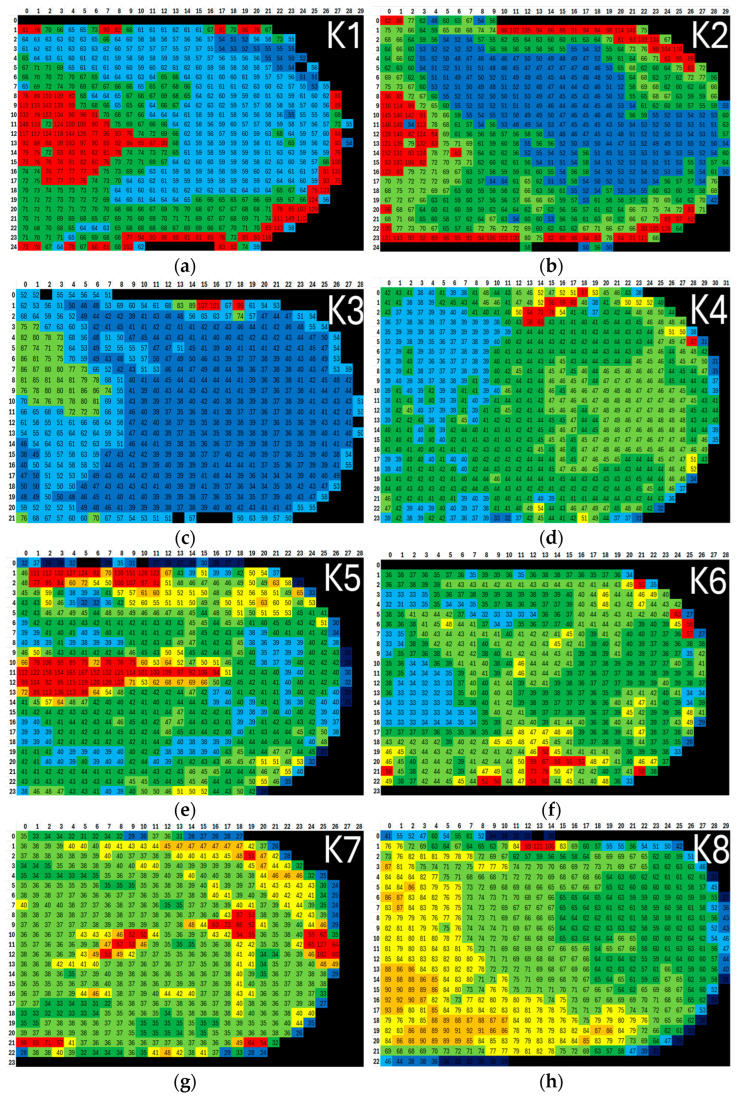
The resulting maps resolve the distribution and average grayscale value of each image. The images are labeled (**a**–**h**), corresponding to the sequential numbering of the samples from K1 to K8, with K1 represented by (**a**) and K8 by (**h**).

The mosaic images grouped by color spectrum and corresponding grayscale values are shown in Figure 9. Each sample has an individually generated spectrum. For sample K1, five color groups were created, with the red and black groups excluded from the analysis. Three procedures were set: dark blue, light blue, and dark green. Sample K2 was also divided into five color groups, with the red and black groups excluded, with the procedures including dark blue, dark green, and light green. Sample K3 was divided into four color groups, with the red group excluded, and three procedures were set. Sample K4 had four procedures, with the red and black groups excluded. For sample K5, seven color groups and five procedures were used, with the red and black groups again excluded. Sample K6 included four procedures, with the red and black groups excluded. For sample K7, four procedures were set, and all other color groups excluded. The same procedure settings and exclusion of other color groups were also applied to sample K8.

When the defined procedures were applied to individual mosaics, Regs files were generated. Each Regs file, exported as a *.csv file from AxioVision software, contained detailed information for every frame within a given sample. Specifically, it contained how many cells and borides were on each image, as well as information about their size, as shown in Table 6. The number of these files corresponded to the number of procedures created for each mosaic. Thus, for example, three Regs files were created for the K6 sample. These files contained a large amount of data. For example, the Regs file for the light blue procedure of the K6 sample was 498,105 kB in size. The Flds file contains overall information for each frame, one by one, i.e., the number of lines in this file corresponded to the number of frames in the respective mosaic. For example, for the K6 sample, the size of this file is 83 kB.

To compare the average cell sizes (grain areas) at various depths, three different measurement approaches were employed (Manual, Auto 1, and Auto 2). The comparison was conducted starting from depth h0, which corresponded to the core of the sample. The final depth values at h4 varied depending on the sample width, which was influenced by defects, deformations, and impurities at the sample edges. For samples K2, K3, K4, K7, and K8, the depths h_0_ to h_4_ were identical, with h_4_ measuring 4535 μm. For samples K1 and K6, the same h_4_ value (5.669 μm) was used; however, they differed in depths h_1_ to h_3_ due to impurities that hindered the analysis. Completely different depths were observed on sample K5 compared to the other samples. The results of the comparison are presented in Table 7.

For sample K1, manual measurement at depth h_0_ revealed that the average grain size was 347 μm^2^. This represents the second-highest measured value at this depth. The highest value in manual measurement is 351 μm^2^ for sample K4. It is assumed that the average grain size values decrease from the core of the sample towards its edge. Based on the obtained results, the average grain size values for sample K1 appears to be similar using all three approaches. In the manual measurements, a decrease is evident in all observed depths (h_0_–h_4_). For the Auto 1 method, a decrease in the average grain size appears to be observed in the depth range h_0_–h_2_, followed by stagnation. A similar trend is observed when using the Auto 2 method.

For sample K2, it appears that from depth h_0_ to h_1_, there is an increase in the average grain size in the manual measurements. In contrast, for Auto 1 and Auto 2 methods, the average grain sizes at these depths seem to remain the same. From depth h_2_ to h_4_, a decrease is observed across all methods.

For sample K3, the manual measurements show a continuous decrease in the average grain size. However, with the Auto 1 and Auto 2 methods, an increase in the average grain size is observed from depth h_0_ to h_1_. From depth h_2_ to h_4_, both Auto 1 and Auto 2 show a decrease.

For sample K4, all three methods indicate an increase in the average grain size from depth h_0_ to h_1_, followed by a decrease from h_2_ to h_4_.

For sample K5, both the manual measurement and the Auto 1 method show an increase in the average grain size from depth h_0_ to h_2_, followed by stagnation. For the Auto 2 method, an increase is observed from h_0_ to h_2_, a decrease at depth h_3_, and another increase at depth h4.

For sample K6, both the manual measurement and the Auto 1 method show a continuous decrease in the average grain size. In the Auto 2 method, a decrease is observed from depth h_0_ to h_3_, followed by an increase at depth h_4_.

For sample K7, the manual measurement shows an increase from depth h_0_ to h_1_, followed by a decrease from h_2_ to h_4_. The Auto 1 method indicates a continuous decrease in the average grain size across all depths from the core to the edge of the sample. In the Auto 2 method, a decrease is observed from depth h_0_ to h_3_, followed by an increase at depth h_4_.

For sample K8, the manual measurement indicates a decrease from depth h_0_ to h_2_. At depth h_3_, there is an observed increase in the average grain size, followed by a decrease at h4. In the Auto 1 method, an increase is observed from depth h_0_ to h_2_, followed by a decrease. The Auto 2 method shows an increase from depth h_0_ to h_3_, followed by a decrease at h_4_.

**Figure 10:**
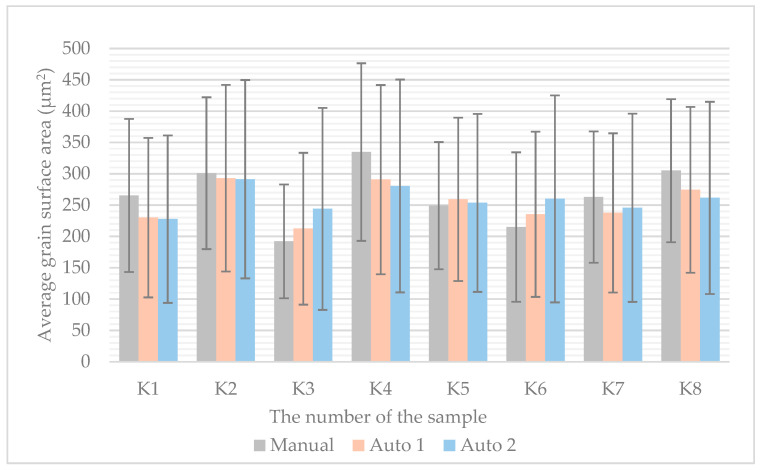
The average grain size values across the entire sample for individual specimens, obtained from measurements using the Manual, Auto 1, and Auto 2 methods, are presented for samples K1 to K8, where K1 corresponds to (**a**) and K8 to (**h**).

The average grain size values across the entire sample for individual specimens are presented in Figure 10. Values obtained from manual measurements are shown in gray, Auto 1 measurements are depicted in orange, and Auto 2 measurements are represented in blue. The figure clearly illustrates the standard deviations in grain size for different measurement approaches.

The smallest difference in grain size between the manual and automatic methods was observed in sample K2. For sample K1, it is evident that the average grain size across the sample is nearly identical for Auto 1 and Auto 2, with a difference of 2.4 μm^2^. An even closer match is observed for sample K2, with a difference of 1.6 μm^2^. For sample K5, the difference is 5.6 μm^2^. The greatest similarity across all measurement approaches was observed for samples K7 and K8. Additionally, similarities were noted between K2 and K4, as well as between K1 and K6. A more detailed description of the results is provided in the statistical analysis.

To assess the grain size and its change from the core (represented by h_0_) closest to the edge of the sample (represented by h_4_), a linear regression was performed on data obtained by three different measurement methods: manual from five frames at each depth h_0_ to h_4_ (Table 8) and automatic Auto 1 (Table 9) and automatic Auto 2 on all frames of the whole sample at each depth (Table 10). Table 8 to Table 10 contain the parameters intercept (the value of grain size in the core of the sample) and slope (the rate of change in grain size towards the surface). The *p*-values for both parameters show that the differences are statistically significant (*p* < 0.05).

The results of sample K1 show that the average grain size (determined as surface area in μm^2^) value found by manual measurement of five frames is 265 μm^2^, and the average grain size found by automated measurement of the area of these same five frames using Auto 2 is 228 μm^2^ (evident from Figure 10). The average grain size determined by automated area measurement with Auto 2 for the whole sample is 183 μm^2^ (evident from Figure 10). It was also found that there is a change in grain size in the direction of heat dissipation during casting. By manual measurement and interleaving the values with a straight line, a gradient of −0.0198 was found, which according to the statistical evaluation is not significant (shown in Table 8). By the Auto 1 measurement, a gradient of −0.022 ± 0.008 was found, which was also not statistically significant (shown in Table 9). In contrast, the gradient of the Auto 2 measurement was −0.013 ± 0.002, which was statistically significant (shown in Table 10).

The results of the K2 sample show that the average grain size (determined as surface area in μm^2^) determined by manual measurement of the five frames is 301 μm^2^, while the average grain size determined by automated area measurement of these five frames using Auto 2 is 291 μm^2^ (evident from Figure 10). The average grain size determined by automated area measurement with Auto 2 for the whole sample is 196 μm^2^ (evident from Figure 10). It was also found that there is a change in grain size in the direction of heat dissipation during casting. By manual measurement and interleaving the values with a straight line, a gradient of −0.0293 was found, which according to the statistical evaluation is significant (shown in Table 8). Using the Auto 1 method, a gradient of −0.03 ± 0.009 was obtained, which did not prove to be statistically significant (shown in Table 9). By Auto 2 measurement and interleaving the values with a straight line, a gradient of −0.0192 ± 0.003 was found, which was also statistically significant (shown in Table 10).

The results of sample K3 show that the average grain size value determined by manual measurement of five frames is 192 μm^2^, and the average grain size determined by automated measurement of the area of these five samples using Auto 2 is 244 μm^2^ (evident from Figure 10). The average grain size determined by the automated Auto 2 area measurement for the whole sample is 233 μm^2^ (evident from Figure 10). It was also found that there is a change in grain size in the direction of heat dissipation during casting. By manual measurement and interleaving the values with a straight line, a gradient of −0.0083 was found, which according to the statistical evaluation is significant (shown in Table 8). Using the Auto 1 measurement, a gradient of −0.011 ± 0.006 was obtained, which did not prove to be statistically significant (shown in Table 9). By Auto 2 measurement and interleaving the values with a straight line, a gradient of −0.0134 ± 0.003 was found, which was also statistically significant (shown in Table 10).

The results of sample K4 show that the average grain size value determined by manual measurement of the five frames is 335 μm^2^, and the grain size determined by automated measurement of the area of these five samples using Auto 2 is 281 μm^2^ (evident from Figure 10). The grain size determined by the automated Auto 2 area measurement for the whole sample is 252 μm^2^ (evident from Figure 10). It was also found that there is a change in grain size in the direction of heat dissipation during casting. By manual measurement and interleaving the values with a straight line, a gradient of −0.0258 was found, which according to the statistical evaluation is significant (shown in Table 8). Using the Auto 1 measurement, a gradient of −0.025 ± 0.006 was obtained, which also proved to be statistically significant (shown in Table 9). By Auto 2 measurement and interleaving the values with a straight line, a gradient of −0.0211 ± 0.001 was found, which was also statistically significant (shown in Table 10).

The results of sample K5 show that the average grain size value determined by manual measurement of the five frames is 249 μm^2^, and the grain size determined by automated measurement of the area of these five samples using Auto 2 is 254 μm^2^ (evident from Figure 10). The grain size determined by the automated Auto 2 area measurement for the whole sample is 270 μm^2^ (evident from Figure 10). It was also found that there is a change in grain size in the direction of heat dissipation during casting. By manual measurement and interleaving the values with a straight line, a gradient of 0.0031 was found, which according to the statistical evaluation is not significant (shown in Table 8). By the Auto 1 measurement, a gradient of −0.014 ± 0.01 was found, which was also not statistically significant (shown in Table 9). By Auto 2 measurement and interleaving the values with a straight line, a gradient of −0.007 ± 0.002 was found, which was statistically significant (shown in Table 10).

The results of sample K6 show that the average grain size value determined by manual measurement from five frames is 215 μm^2^, and the grain size determined by automated area measurement of these five samples using Auto 2 is 260 μm^2^ (evident from Figure 10). The grain size determined by the automated Auto 2 area measurement for the whole sample is 251 μm^2^ (evident from Figure 10). It was also found that there is a change in grain size in the direction of heat dissipation during casting. By manual measurement and interleaving the values with a straight line, a gradient of −0.0215 was found, which according to the statistical evaluation is significant (shown in Table 8). Using the Auto 1 measurement, a gradient of −0.031 ± 0.003 was obtained, which also proved to be statistically significant (shown in Table 9). By Auto 2 measurement and interleaving the values with a straight line, a gradient of −0.012 ± 0.005 was found, which was also statistically significant (shown in Table 10).

The results of sample K7 show that the average grain size value determined by manual measurement of the five frames is 263 μm^2^, and the grain size determined by automated measurement of the area of these five samples using Auto 2 is 246 μm^2^ (indicated by the red line in Figure 10). The grain size determined by the automated Auto 2 area measurement for the whole sample is 232 μm^2^ (evident from Figure 10). It was also found that there is a change in grain size in the direction of heat dissipation during casting. By manual measurement and interleaving the values with a straight line, a gradient of −0.0164 was found, which according to the statistical evaluation is significant (shown in Table 8). Using the Auto 1 measurement, a gradient of −0.028 ± 0.002 was obtained, which also proved to be statistically significant (shown in Table 9). By Auto 2 measurement and interleaving the values with a straight line, a gradient of −0.010 ± 0.002 was found, which according to the statistical evaluation is not significant (shown in Table 10).

The results of sample K8 show that the average grain size value determined by manual measurement from five frames is 305 μm^2^, and the grain size determined by automated measurement of the area of these five samples using Auto 2 is 262 μm^2^ (evident from Figure 10). The grain size determined by the automated Auto 2 area measurement for the whole sample is 256 μm^2^ (evident from Figure 10). It was also found that there is a change in grain size in the direction of heat dissipation during casting. By manual measurement and interleaving the values with a straight line, a gradient of 0.0091 was found, which according to the statistical evaluation is not significant (shown in Table 8). By the Auto 1 measurement, a gradient of −0.00012 ± 0.01 was found, which was also not statistically significant (shown in Table 9). By Auto 2 measurement and interleaving the values with a straight line, a gradient of −0.015 ± 0.002 was found, which was statistically significant (shown in Table 10).

**Figure 11:**
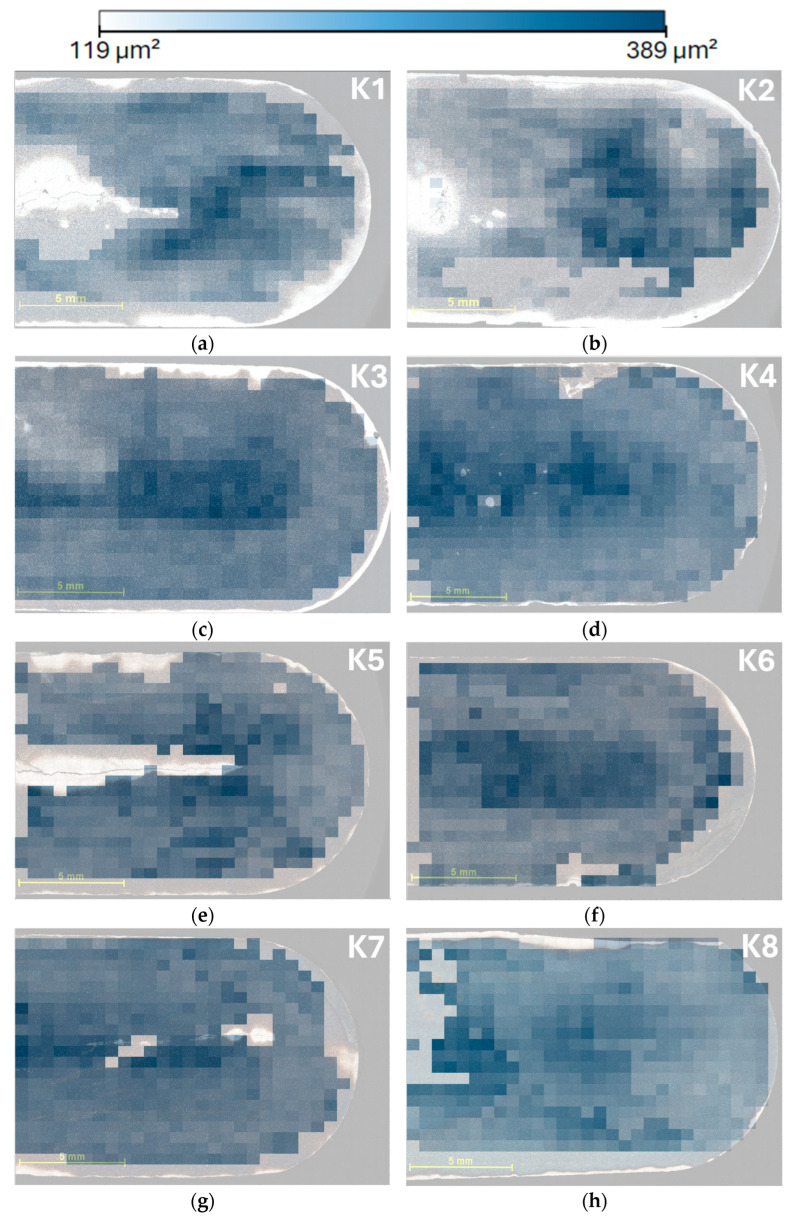
Resulting maps addressing the distribution and average size of cells within individual frames. The images are labeled (**a**–**h**), corresponding to the sequential numbering of the samples from K1 to K8, with K1 represented by (**a**) and K8 by (**h**).

**Figure 12:**
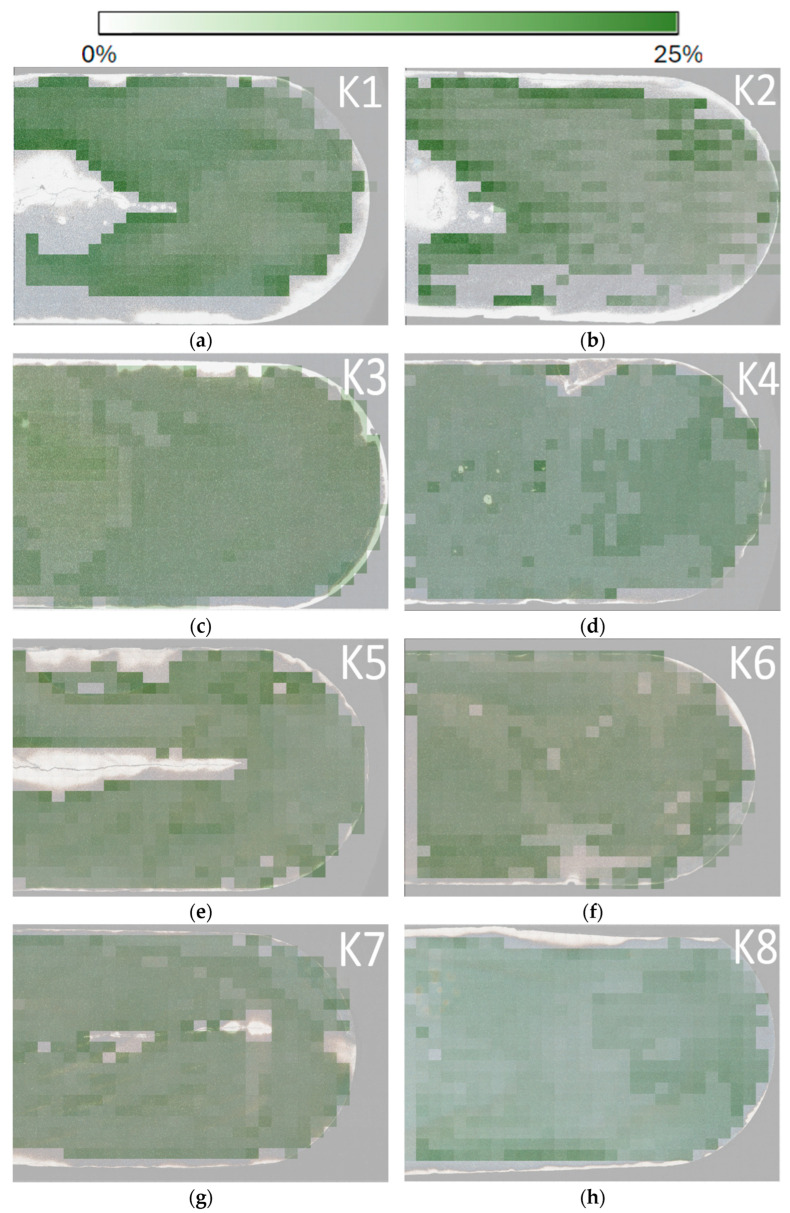
Resulting maps of Phase 2 (borides) representation within each image. The images are labeled (**a**–**h**), corresponding to the sequential numbering of the samples from K1 to K8, with K1 represented by (**a**) and K8 by (**h**).

To visualize the grain size distribution in each sample, maps (Figure 11) were created to show the spatial distribution of cell sizes. In these maps, areas with larger grains are shown in dark blue, indicating a higher concentration of larger grains. Conversely, lighter areas indicate the presence of smaller grains. Frames that were not suitable for images were 119 μm^2^ in size, while the largest grain was 389 μm^2^ in size. The maps were created so that the colors matched for all images and the cell sizes were displayed consistently, allowing for easy comparability between samples. This visual overview makes it easy to identify areas of the sample with higher and lower grain size values. Maps (Figure 12) showing phase fraction, specifically the phase distribution of borides, were also created for all eight samples. These maps clearly show that the lighter areas indicate a lower concentration of Phase 2 (borides), while the darker areas show a higher abundance of this phase, up to 25%. The maps were designed so that the colors were consistent for all samples, allowing for easy comparability between samples. This overview provides a visual representation of the distribution of phases in the material, making it easier to identify areas with different boride concentrations. As with the grain size map, only frames that were suitable for phase composition detection were selected for analysis, and frames unsuitable for this analysis were excluded.

The measurement results of sample K1 show a rapid decrease in grain size from the core (h_0_) to a depth of 1134 μm (h_1_). Thereafter, there is a smaller decrease in grain size towards the surface. This sample shows the most pronounced decrease in cell size between h_0_ and h_1_ of all the samples at the initial depths, which may be influenced by its chemical composition, particularly its low alloying element content. K1 has the lowest intercept among all samples.

For sample K2, the decrease in grain size from the core was less pronounced up to a depth of 2268 μm (h_2_). From this depth onwards, there is a steeper decrease in grain size towards the surface. The results indicate that the gradient of cell size change is more gradual than that of sample K1. Based on this fact, it can be concluded that the increase in chromium content K2 affects the average grain size such that the grain size value increases and the gradient of grain size change is also higher, which was confirmed by both manual and automatic measurements.

For sample K3, the decrease in grain size is uniform across all depths (h_0_ to h_4_). The cell sizes are smaller than those of K1 and K2, and in the case of this sample, only vanadium was added as an alloying element at 0.51, with no chromium added. The grain size gradient is smaller compared to K1, which may be due to the difference in chromium and other alloying elements. This sample shows a more homogeneous microstructure across depths.

Sample K4 combines a larger grain diameter with a more uniform texture compared to the other samples. Sample K4 shows a distinct initial grain size and the steepest gradient of grain size decrease across the entire cross-section. The higher chromium content, together with the presence of vanadium, resulted in a larger initial grain size and a gradual refinement towards the surface. The intercept of this sample was the largest among all samples. The slope was −0.0211 ± 0.001, indicating the steepest decrease in cell size. The high chromium content (0.95%) seems to have a significant effect on the cell size and the gradient of change.

The grain size of sample K5 decreases very slightly from the core to the edge of the sample, indicating an almost homogeneous microstructure. Thus, the absence of vanadium and lower chromium content may contribute to the smaller initial grain size and minimal grain size changes at different depths. The slope was −0.007 ± 0.002, indicating the lowest decrease among all samples.

A gradual decrease in grain size was observed for sample K6, similar to sample K4, but the gradient of decrease was less pronounced. The chemical composition, characterized by a higher chromium content, could contribute to the larger average grain size, with a more moderate change towards the surface.

Sample K7 shows very similar behavior to sample K5. The grain size gradually decreases, but the gradient of change is minimal. This homogeneous microstructure confirms the influence of a similar chemical composition. The slope is −0.010 ± 0.002, indicating a slight gradient in grain size variation. The results are very similar to sample K5, which corresponds to a nearly identical chemical composition.

A slight decrease in grain size was observed for sample K8 compared to K6 at various depths. This sample is characterized by a higher average grain size (determined as surface area in μm^2^). This sample has a chemical composition similar to K6 and shows similar cell size trends.

## 4. Discussion and Conclusions

Grain size and microstructural characteristics have been the subject of numerous studies, often focusing on the quantification of microstructure using both manual and automated methods [19,21,27]. However, these studies typically focus on the analysis of individual selected (ideal) images from the entire sample. This limits the possibility of obtaining a comprehensive overview of microstructural changes. Our study introduces another approach for rapid, accessible, and straightforward analysis and processing of image data across large sample areas. This enables a more detailed examination of the grain size and distribution in the materials. Although this approach provides valuable insights, it is necessary to address the limitations associated with the analysis of grain size and its distribution in Fe-B-C alloys using optical microscopy and image analysis. The first limitation arises from the material itself, as the chemical composition must allow for the formation of a hypoeutectic structure [28]. In addition, the microstructure must exhibit a cellular or at least partially cellular morphology, which typically corresponds to at least 0.2 wt. % boron, depending on the presence of other alloying elements [29]. The chemical composition in our study was obtained through OES analysis and further verified by EDS analysis excluding carbon. Further heat treatment of the sample is essential to prevent the formation of ferrite–pearlite. For our purposes, the most suitable method has proven to be quenching at 880 °C for 20 min, followed by rapid cooling of the sample in a water bath. Another important consideration is the need to use color etching techniques for effective visualization of the microstructure. This method is indispensable for grain resolution and the assessment of grain distribution. Although etching techniques may require additional care when applied to such large samples, basic microstructural characteristics such as grain size and grain size gradient can be determined by whole-area analysis over a sufficiently large sample area [30,31,32]. In 2018, Campbell et al. [30] utilized etching techniques such as Beraha’s reagent to enhance grain boundaries and Murakami’s etchant for selective highlighting of carbide phases. Additionally, they applied destabilization annealing at temperatures close to 950 °C, which resulted in the precipitation of secondary carbides, and employed various cooling rates to control the final microstructure. However, this procedure seems to be more effective with higher chromium addition [33]. In our study, the alloys contained at the most 0.38 wt. % B and 1.06 wt. % Cr, and boride formation occurred directly at the boundaries; therefore, it was not necessary to use pre-etching in this case. In our study, four approaches were compared to identify the most suitable method for visualizing microstructures and clearly delineating the boundaries between individual phases. The samples were prepared using different etching techniques, including unpolished structures, microstructures etched with a 2% Nital solution, quenched microstructures etched with Nital, and quenched microstructures etched with Klemm I color etchant. Among these, etching with Klemm I color etchant proved to be the most appropriate technique.

This study focused on the analysis of grain size and its distribution in Fe-B-C alloys with low boron content. In materials engineering, attention is primarily directed towards alloys with single-phase structures [34,35], with less focus given to multiphase alloys. Fe-B-C alloys have been studied, but typically only in the hypereutectoid range and with high boron content [8,36,37]. Insufficient attention has been given to the study of boron distribution in the hypoeutectoid Fe-B-C alloys with low boron concentrations, especially when analyzing the entire cross-section of the material.

Eight Fe-B-C alloy samples (K1–K8) with varying chemical compositions of specific elements (B, Cr, W, V) were analyzed using both manual and automated measurement methods. The manual method involved precise measurements at five depth levels (h_0_–h_4_) on selected frames, while automated methods employed image analysis across the entire sample area using software with procedures modified to align results with manual measurements.

The individual mosaic images were divided into color groups according to grayscale. This technique is commonly employed in metallography for image analysis, particularly in automated methods for grain boundary segmentation. This approach enhances the accuracy of grain boundary identification and facilitates subsequent steps, such as grain size measurement [38,39,40]. Although manual measurement is challenging and dependent on observer subjectivity, it provides a good basis for validating automatic methods [41,42,43]. The automated measurements, due to the larger range of data, clearly revealed a trend of decreasing cell size from the nucleus to the surface in all samples, whereas the manual measurements confirmed this decrease only in some of them (K2, K3, K4, K6, K7). This may be due to the greater subjectivity of manual measurements or the limited number of frames analyzed, which raises the question of whether the manual method can be considered sufficiently reliable for analyses with high demands on statistical accuracy. On the other hand, manual measurement allowed more precise control over the selection of images and provided a basic insight into the influence of alloying elements, the gradient of cell size change, and phase representation.

Statistical analysis of the automated method in this study revealed significant differences in the grain size gradient among the samples, indicating the influence of chemical composition (regarding elements such as Cr, V, and W) on grain size distribution. Samples with higher chromium content (e.g., K2, K4, K8) show more pronounced microstructural changes, suggesting that a higher proportion of alloying elements causes both a larger cell size and a more pronounced gradient. However, this effect may not be universal. The homogeneous microstructure of samples with lower chromium content (e.g., K5) shows that the mechanism depends not only on the chromium concentration but also on the interaction with other elements such as vanadium and tungsten. Samples with higher tungsten content (e.g., K5, K7) had a more uniform grain size distribution from the core to the surface. The results further confirmed that microstructural homogeneity decreases with the addition of alloying elements such as chromium and vanadium, while tungsten contributes to more stable grain size gradients.

The role of automated methods, which provide comprehensive statistical insight, remains debatable, but at the cost of less control over the analysis process. These differences highlight the need to combine the two approaches for the most accurate assessment of microstructural changes.

In conclusion, the integration of manual and automated techniques, supported by statistical evaluation and mosaic image analysis, provided a robust framework for characterizing grain size distributions and microstructural gradients in the studied hypoeutectic Fe-B-C alloy samples. These findings emphasize the importance of tailoring chemical compositions to achieve specific microstructural properties and offer valuable insights for materials design and optimization. The combination of manual and automated measurements not only identified basic trends but also confirmed them with statistical significance. Automated methods proved particularly suitable for analyzing complex microstructures due to their ability to handle larger datasets with higher accuracy. Ultimately, the use of these complementary approaches enabled a reliable and detailed understanding of grain size variations from the core to the edge of individual Fe-B-C alloy samples.

## Figures and Tables

**Table 1:** The chemical composition (wt. %) of the ferroalloys (obtained from the Commexim Group’s a.s. attestations, verified by EDS analysis without carbon) used for the preparation of the samples.

Ferroalloys	C	Si	P	Cr	Al	N	B	W	V	Fe
FeB	0.31	0.43	0.037	-	0.07	-	18.49	-	-	Bal.
FeCr LC	0.06	1.32	0.02	69.40	-	0.04	-	-	-	Bal.
FeW	0.36	0.35	0.02	-	-	-	-	76.64	-	Bal.
FeV	0.25	1.25	0.033	-	1.73	-	-	-	80.73	Bal.

**Table 2:** Chemical compositions (wt. %) of steel C35 used for the preparation of the samples.

Steel Bars	C	Si	Mn	P	S	Cr	Ni	Cu	Al	Fe
C35	0.37	0.21	0.70	0.005	<0.150	0.05	0.060	0.179	0.031	Bal.

**Table 3:** The chemical compositions (wt. %) of cast steels for the creation of the samples K1 to K8.

Sample	C	Si	Mn	P	S	Cr	Ni	Cu	Al	B	V	W	Fe
K1	0.35	0.26	0.65	0.006	<0.15	0.52	0.06	0.16	0.02	0.35	-	-	Bal.
K2	0.36	0.28	0.62	0.006	<0.15	1.06	0.06	0.16	0.02	0.36	-	-	Bal.
K3	0.33	0.25	0.61	0.005	<0.15	0.05	0.05	0.16	0.01	0.35	0.51	-	Bal.
K4	0.33	0.23	0.65	0.009	<0.15	0.95	0.06	0.17	0.02	0.38	0.52	-	Bal.
K5	0.35	0.23	0.65	0.006	<0.15	0.47	0.06	0.16	0.01	0.36	-	0.51	Bal.
K6	0.37	0.26	0.65	0.007	<0.15	1.02	0.06	0.16	0.02	0.38	-	0.49	Bal.
K7	0.35	0.24	0.65	0.007	<0.15	0.58	0.06	0.17	0.02	0.34	-	0.53	Bal.
K8	0.35	0.25	0.65	0.006	<0.15	1.07	0.06	0.16	0.02	0.37	-	0.43	Bal.

**Table 4:** Color differentiation of individual procedures according to shades of gray for sample K6.

Cell Value	Color	Procedure	Setup Pictures with Cell Value
≤25			
(25;30⟩			
(30;35⟩		Procedure 1	403 (32), 471 (34)
(35;40⟩		Procedure 2	424 (36), 105 (39)
(40;45⟩		Procedure 3	238 (42), 585 (44)
(45;50⟩		Procedure 4	302 (46), 629 (49)
>50			

**Table 5:** The metadata parameters of individual mosaics for all examined samples (K1–K8).

Sample	Number of Rows (m)	Number of Columns (n)	Number of Frames	File Size [kB]	Image Width [px]	Image Height [px]
K1	25	30	750	22,285,792	66,774	46,466
K2	25	30	750	22,285,792	66,774	46,466
K3	22	29	638	18,957,788	64,557	40,914
K4	24	32	768	22,820,644	71,210	44,615
K5	24	29	696	20,681,220	64,557	44,615
K6	24	29	696	20,681,220	64,557	44,615
K7	24	29	696	20,681,220	64,557	44,615
K8	23	30	690	20,502,928	66,774	42,765

**Table 6:** Regs file with input values used in the algorithm.

ImageName	Image Phase Name	Densitometric Mean (Gray)	Densitometric Minimum (Gray)	Densitometric Maximum (Gray)	Area (px^2^)
K6_200x_p001.tif	Phase 1	22	7	39	13,432
K6_200x_p001.tif	Phase 1	23	8	54	42,191
…	...	...	...	...	...
K6_200x_p001.tif	Phase 2	64	42	81	39
K6_200x_p001.tif	Phase 2	58	47	73	17

**Table 7:** The resulting values from measurements conducted using manual and automatic approaches for each sample at five different depths.

		Depth	Manual	Auto 1	Auto 2			Depth	Manual	Auto 1	Auto 2
		[μm]	[μm^2^]	[μm^2^]	[μm^2^]			[μm]	[μm^2^]	[μm^2^]	[μm^2^]
K1	h_0_	0	347	331	328	K5	h0	0	263	247	234
	h_1_	1134	270	239	225		h1	850	291	290	245
	h_2_	2268	247	200	201		h2	1984	289	328	292
	h_3_	4535	235	203	202		h3	3827	227	228	224
	h_4_	5669	228	177	182		h4	4819	176	203	273
K2	h_0_	0	293	333	334	K6	h0	0	332	341	335
	h_1_	1134	332	337	343		h1	1701	262	283	307
	h_2_	2268	317	323	314		h2	3401	189	207	215
	h_3_	3401	315	283	254		h3	5102	174	191	208
	h_4_	4535	248	189	212		h4	5669	118	155	235
K3	h_0_	0	219	217	256	K7	h0	0	304	313	292
	h_1_	1134	210	236	264		h1	1134	283	256	261
	h_2_	2268	208	237	243		h2	2268	281	239	237
	h_3_	3401	176	203	232		h3	3401	238	198	221
	h_4_	4535	148	169	225		h4	4535	208	182	218
K4	h_0_	0	351	325	316	K8	h0	0	330	251	232
	h_1_	1134	380	342	334		h1	1134	305	270	238
	h_2_	2268	354	307	298		h2	2268	347	318	287
	h_3_	3401	312	256	237		h3	3401	294	294	296
	h_4_	4535	277	224	218		h4	4535	249	239	255

**Table 8:** Statistical evaluation of manual measurement data determined by regression analysis.

Sample	Intercept	*p*-Value	Slope	*p*-Value	R^2^	*p*-Value
K1	281.6	0.002	−0.020	0.104	0.640	0.104
K2	357.8	<0.001	−0.029	0.020	0.873	0.020
K3	262.9	<0.001	−0.008	0.029	0.838	0.029
K4	339.2	<0.001	−0.026	0.029	0.839	0.029
K5	246.6	0.002	0.003	0.721	0.049	0.721
K6	328.4	<0.001	−0.022	0.045	0.787	0.045
K7	283.0	<0.001	−0.016	0.010	0.920	0.010
K8	240.9	0.001	0.009	0.314	0.327	0.314

**Table 9:** Statistical evaluation of Auto 1 measurement data determined by regression analysis.

Sample	Intercept	*p*-Value	Slope	*p*-Value
K1	289.1 ± 27.5	0.002	−0.022 ± 0.008	0.073
K2	361.2 ± 10.7	<0.001	−0.030 ± 0.009	0.054
K3	238.2 ± 17.27	<0.001	−0.011 ± 0.006	0.165
K4	348.4 ± 16.9	<0.001	−0.025 ± 0.006	0.025
K5	291.5 ± 34.4	0.003	−0.014 ± 0.010	0.319
K6	335.0 ± 12.8	<0.001	−0.031 ± 0.003	0.003
K7	301.3 ± 9.4	<0.001	−0.028 ± 0.002	0.003
K8	274.4 ± 28	0.002	−0.0001 ± 0.010	0.999

**Table 10:** Statistical evaluation of Auto 2 measurement data determined by regression analysis.

Sample	Intercept	*p*-Value	Slope	*p*-Value
K1	226.9 ± 7.1	<0.001	−0.013 ± 0.002	<0.001
K2	268.8 ± 10.7	<0.001	−0.019 ± 0.003	<0.001
K3	283.5 ± 8.9	<0.001	−0.013 ± 0.003	<0.001
K4	312.7 ± 4.9	<0.001	−0.021 ± 0.001	<0.001
K5	311.1 ± 8.3	<0.001	−0.007 ± 0.002	0.008
K6	284.1 ± 13.7	<0.001	−0.012 ± 0.005	0.030
K7	267.3 ± 6.1	<0.001	−0.010 ± 0.002	<0.001
K8	321.8 ± 5.3	<0.001	−0.015 ± 0.002	<0.001

## Data Availability

The original contributions presented in this study are included in the article. Further inquiries can be directed to the corresponding author.
